# Baixa Concordância entre a Classificação da NYHA e as Variáveis do Teste de Exercício Cardiopulmonar em Pacientes com Insuficiência Cardíaca e Fração de Ejeção Reduzida

**DOI:** 10.36660/abc.20210222

**Published:** 2022-03-21

**Authors:** Luiz Eduardo Fonteles Ritt, Rebeca Sadigursky Ribeiro, Isabela Pilar Moraes Alves de Souza, João Victor Santos Pereira Ramos, Daniel Sadigursky Ribeiro, Gustavo Freitas Feitosa, Queila Borges de Oliveira, Ricardo Stein, Eduardo Sahade Darzé

**Affiliations:** 1 IDOR Hospital Cárdio Pulmonar Salvador BA Brasil Instituto D’or de Pesquisa e Ensino (IDOR), Hospital Cárdio Pulmonar, Salvador, BA - Brasil; 2 Escola Bahiana de Medicina e Saúde Pública Salvador BA Brasil Escola Bahiana de Medicina e Saúde Pública, Salvador, BA - Brasil; 3 Hospital de Clínicas de Porto Alegre Universidade Federal do Rio Grande do Sul Porto Alegre RS Brasil Hospital de Clínicas de Porto Alegre, Universidade Federal do Rio Grande do Sul, Porto Alegre, RS - Brasil

**Keywords:** Insuficiência Cardíaca, Prognóstico, Teste de Esforço

## Abstract

**Fundamento:**

A classificação funcional da New York Heart Association (NYHA) é o sistema de classificação mais utilizado para a insuficiência cardíaca (IC), enquanto o teste de exercício cardiopulmonar (TECP) é o padrão ouro para a avaliação do estado funcional na IC.

**Objetivo:**

Analisar a correlação e a concordância entre as classes da NYHA e as variáveis do TECP.

**Métodos:**

Foram selecionados pacientes com IC com indicação clínica para TECP e fração de ejeção (FE) < 50%. A correlação (coeficiente de Spearman) e a concordância (kappa) entre a classificação da NYHA e as classificações baseadas no TECP foram analisadas. Um valor de p < 0,05 foi considerado significativo.

**Resultados:**

No total, foram incluídos 244 pacientes no estudo. A idade média foi de 56±14 anos, e a FE média foi de 35,5%±10%. A distribuição de pacientes de acordo com a classificação da NYHA foi a seguinte: classe I (31,2%), classe II (48,3%), classe III (19,2%) e classe IV (1,3%). A correlação (r) entre as classes da NYHA e de Weber foi de 0,489 (p < 0,001), e a concordância foi de 0,231 (p < 0,001). A correlação (r) entre as classes da NYHA e ventilatórias (inclinação da ventilação minuto/produção de dióxido de carbono [VE/VCO_2_]) foi de 0,218 (p < 0,001), e a concordância foi de 0,002 (p = 0,959). A correlação de Spearman entre as classes da NYHA e do escore TECP foi de 0,223 (p = 0,004), e a concordância kappa foi de 0,027 (p = 0,606).

**Conclusão:**

Foi identificada uma associação moderada entre as classes da NYHA e de Webber, embora a concordância tenha sido baixa. As classes ventilatórias (inclinação VE/VCO_2_) e do escore TECP apresentaram uma associação fraca e uma baixa concordância com as classes da NYHA.

## Introdução

Embora seja uma doença progressiva, a insuficiência cardíaca (IC) não apresenta um curso linear. As hospitalizações por IC descompensada são fatores independentes de prognóstico. Modelos de predição de risco e escores prognósticos determinarão a necessidade de escalonar estratégias terapêuticas específicas, como alterações do medicamento, terapia de ressincronização cardíaca, cardioversor-desfibrilador implantável, dispositivo de assistência ventricular e transplante cardíaco.^1^

A classificação da New York Heart Association (NYHA) é um instrumento de estratificação funcional para IC simples, de baixo custo e bem conhecido, com valor prognóstico.^2 , 3^ Divide os pacientes em quatro grupos diferentes, de acordo com a gravidade da dispneia e as limitações da atividade física autorrelatadas.^2 , 3^ No entanto, visto que a classe funcional da NYHA depende do autorrelato de sintomas, é influenciada pela subjetividade de cada paciente.^4 , 5^

Em contrapartida, o estado funcional é avaliado objetivamente pelo teste de exercício cardiopulmonar (TECP), um instrumento prognóstico considerado o padrão ouro para a avaliação da IC.^6 , 7^ Nesse contexto, diretrizes importantes definem o TECP como uma recomendação de classe I para o transplante cardíaco e de classe IIa para a prescrição de exercícios.^6 , 7^

Normalmente, a avaliação prognóstica do TECP é baseada nas medidas do pico de consumo de oxigênio (VO_2peak_).^8 , 9^ No entanto, outras variáveis, como a inclinação da ventilação minuto/produção de dióxido de carbono (VE/VCO_2_), recuperação da frequência cardíaca em 1 minuto (HRR_1_), inclinação da eficiência de consumo de oxigênio (OUES), pressão parcial do dióxido de carbono ao final da expiração (PetCO_2_) e ventilação periódica, têm demonstrado um valor prognóstico independente e incremental ao VO_2peak_ na IC.^10^ Com base nessas variáveis, classificações prognósticas específicas foram validadas, como as classes de Weber (VO_2peak_), as classes ventilatórias (inclinação VE/VCO_2_) e o escore TECP (uma combinação de VO_2peak_, inclinação VE/VCO_2_, HRR_1_, OUES e PetCO_2_).^11^

Embora o sistema de classificação da NYHA seja amplamente utilizado, há poucos estudos correlacionando as classes da NYHA com o prognóstico de IC ou as variáveis do TECP.^14 , 15^ Uma revisão sistemática recentemente comparou as classificações da NYHA e as variáveis do TECP. A variável comum em todos os estudos analisados foi o VO_2peak_, embora com muita heterogeneidade.^14^ O objetivo do presente estudo foi avaliar a correlação e a concordância entre a classificação da NYHA para IC e as classificações funcionais baseadas no TECP, isto é, as classes de Weber, as classes ventilatórias e o escore TECP.^11^

## Métodos

O presente estudo transversal recrutou consecutivamente pacientes submetidos a TECP para avaliação da IC. Os critérios de inclusão foram os seguintes: 1) idade ≥ 18 anos; 2) diagnóstico confirmado de IC com fração de ejeção (FE) < 50%; e 3) indicação clínica para TECP entre 2009 e 2019. Os critérios de exclusão foram doença pulmonar obstrutiva crônica moderada a grave, hipertensão pulmonar e/ou fibrose ou anemia sintomática.

Os dados demográficos e as variáveis do TECP foram coletados junto a informações clínicas e testes complementares relevantes (eletrocardiograma em repouso de 12 derivações e ecocardiografia com Doppler dos últimos 3 meses). O TECP foi limitado aos sintomas e realizado com esforço máximo através de um protocolo de rampa em uma esteira (Micromed Centurion 300, São Paulo, Brasil), utilizando um ergoespirômetro respiração a respiração Cortex 3b (Cortex Inc., Leipzig, Alemanha). A calibração de gás de dois pontos foi realizada antes dos testes. Todas as técnicas foram realizadas de acordo com as diretrizes atuais, e um médico certificado em nível nacional foi responsável por cada teste.^10^

Todos os TECPs foram conduzidos pelo mesmo médico, um cardiologista especializado em TECP. Antes da realização do teste, o cardiologista determinou a classe da NYHA de cada paciente de acordo com a limitação da atividade física autorrelatada: (I) sem limitação; (II) pouca limitação; (III) limitação acentuada; ou (IV) incapaz de realizar qualquer atividade física sem desconforto.^16^ Em seguida, com base nas variáveis do TECP, os pacientes foram categorizados em classes de Weber, classes ventilatórias ou classes do escore TECP de acordo com os resultados do teste.^11^

A classificação de Weber categoriza os pacientes de acordo com o VO_2peak_, da seguinte maneira: (A) VO_2_ > 20 mL.kg^-1^.min^-1^; (B) VO_2_ 16-20 mL.kg^-1^.min^-1^; (C) VO_2_ 10-15 mL.kg^-1^.min^-1^; ou (D) VO_2_ < 10 mL.kg^-1^.min^-1^.^12^ As classes ventilatórias utilizam a inclinação VE/VCO_2_: (I) VE/VCO_2_ ≤ 29,9; (II) VE/VCO_2_ 30-35,9; (III) VE/VCO_2_ 36-44,9; ou (IV) VE/VCO_2_ ≥ 45.^13^ O escore TECP foi calculado para cada paciente com base na soma das respostas anormais, da seguinte maneira: VE/VCO_2_ ≥ 34 (7 pontos); HRR_1_ ≤ 6 bpm (5 pontos); OUES ≤ 1,4 (3 pontos); PetCO_2_ < 33 mm Hg (3 pontos); e VO_2peak_ ≤ 14 mL.kg^-1^.min^-1^ (2 pontos).^11 , 15^ Na sequência, o escore foi dividido em quartis: (I) 0-5; (II) 6-10; (III) 10-15; e (IV) > 15.^11^

### Análise estatística

As análises estatísticas foram realizadas no *software* SPSS versão 17.0 (SPSS Inc., Chicago, IL, EUA). As variáveis contínuas foram apresentadas como média e desvio padrão para a distribuição paramétrica ou como mediana e intervalo interquartil para a distribuição não paramétrica. O teste de normalidade de Kolmogorov-Smirnov e as análises de histograma foram utilizados para determinar a distribuição. As variáveis categóricas foram apresentadas como números absolutos e proporções. A correlação entre as variáveis foi avaliada através do coeficiente de correlação de Spearman ( *s* ) ou de Pearson ( *p* ), e a concordância foi avaliada através do coeficiente kappa (k). Para todas as análises, um valor de p < 0,05 foi considerado estatisticamente significativo.

O Comitê de Ética em Pesquisa da instituição aprovou o protocolo do estudo. O estudo está em conformidade com todos os regulamentos nacionais e internacionais para pesquisas com seres humanos.

## Resultados

As características dos pacientes estão descritas na Tabela 1 . A amostra incluiu 244 pacientes, principalmente homens (77,9%), com idade média de 56±14 anos. A isquemia foi a etiologia mais frequente (44,4%). A FE média foi de 35,5%±10%. Os pacientes receberam terapia médica otimizada, conforme a seguir: inibidores da enzima conversora da angiotensina ou bloqueadores dos receptores de angiotensina II (86,4%); betabloqueadores (91,4%); antagonistas da aldosterona (57,0%); e diuréticos (53,5%). O VO_2peak_ médio foi de 19,2±6,7 mL.kg^-1^.min^-1^, enquanto a inclinação VE/VCO_2_ média foi de 39±10. A taxa de troca respiratória (RER) média foi de 1,041±0,12 (25% apresentaram RER > 1,10). Todos os testes foram interrompidos pelo critério de esforço, e nenhum foi interrompido prematuramente ou devido a critérios hemodinâmicos, arrítmicos ou isquêmicos. Os pacientes foram distribuídos de acordo com a classificação da NYHA, da seguinte maneira: classe I (31,2%), classe II (48,3%), classe III (19,2%) e classe IV (1,3%) ( Tabela 2 ).

**Tabela 1:** – Características demográficas, clínicas e do teste de exercício cardiopulmonar gerais dos pacientes (n = 244)

Variáveis	
**Idade** (média ± DP)	56±14 anos
**Sexo**	
Masculino, n (%)	190 (77,9)
**Etiologia**	
Isquêmico, n (%)	107 (44,4)
Idiopático, n (%)	56 (23,2)
Viral, n (%)	30 (12,4)
Chagásico, n (%)	18 (7,5)
Outro, n (%)	30 (12,5)
**Comorbidades**	
Hipertensão, n (%)	70 (34,7)
Diabetes melito, n (%)	43 (21,2)
Doença arterial coronariana, n (%)	94 (46,3)
Tabagismo, n (%)	4 (2,0)
**Medicamentos utilizados**	
IECA ou BRA, n (%)	209 (86,4)
Betabloqueador, n (%)	222 (91,4)
ARM, n (%)	138 (57,0)
Diuréticos, n (%)	129 (53,5)
**Dispositivos implantáveis**	
Marca-passo, n (%)	17 (7,0)
TRC e/ou CDI, n (%)	28 (11,5)
**VO**_**2peak**_ **(mL.kg** ^**-1**^ **.min** ^**-1**^ **)** , média ± DP	19,2±6,7
**Porcentagem do VO**_**2peak**_ **previsto (%)** , média ± DP	63±20
**FE (%)** , média ± DP	35,5±10
**RER** , média ± DP	1,041±0,12
**Inclinação VE/VCO**_**2**_ , média ± DP	39,0±10,8
**PetCO**_**2**_ **(mm Hg)** , média ± DP	29,8±4,66
**HRR**_**1**_ , mediana (IIQ)	18,0 (15)
**PAS em repouso** , mediana (IIQ)	120 (10)
**FC em repouso** , mediana (IIQ)	74 (22)

*ARM: antagonistas dos receptores de mineralocorticoides; BRA: bloqueadores dos receptores de angiotensina II; CDI: cardioversor-desfibrilador implantável; DP: desvio padrão; FC: frequência cardíaca; FE: fração de ejeção; HRR_1_: recuperação da frequência cardíaca em 1 minuto; IECA: inibidor da enzima conversora de angiotensina; NYHA: New York Heart Association; PAS: pressão arterial sistólica; PetCO_2_: pressão parcial do dióxido de carbono ao final da expiração; RER: taxa de troca respiratória; TECP: teste de exercício cardiopulmonar; IIQ; intervalo interquartil; TRC: terapia de ressincronização cardíaca; VE/VCO_2_: ventilação minuto/produção de dióxido de carbono; VO_2peak_: pico de consumo de oxigênio.*

**Tabela 2:** – Distribuição da amostra de acordo com classificações objetivas e subjetivas, n (%)

	I	II	III	IV
Classes da NYHA	75 (31,3)	116 (48,3)	46 (19,2)	3 (1,3)
Inclinação VE/VCO_2_	42 (17,2)	70 (28,7)	74 (30,3)	58 (23,8)
Escore TECP	57 (34,7)	61 (37,2)	36 (22,0)	10 (6,1)
	A	B	C	D
Classes de Weber	95 (39)	55 (22,5)	81 (33,2)	13 (5,3)

*CPET: teste de exercício cardiopulmonar; NYHA: New York Heart Association; VE/VCO_2_: ventilação minuto/produção de dióxido de carbono.*

**Figura 1:**
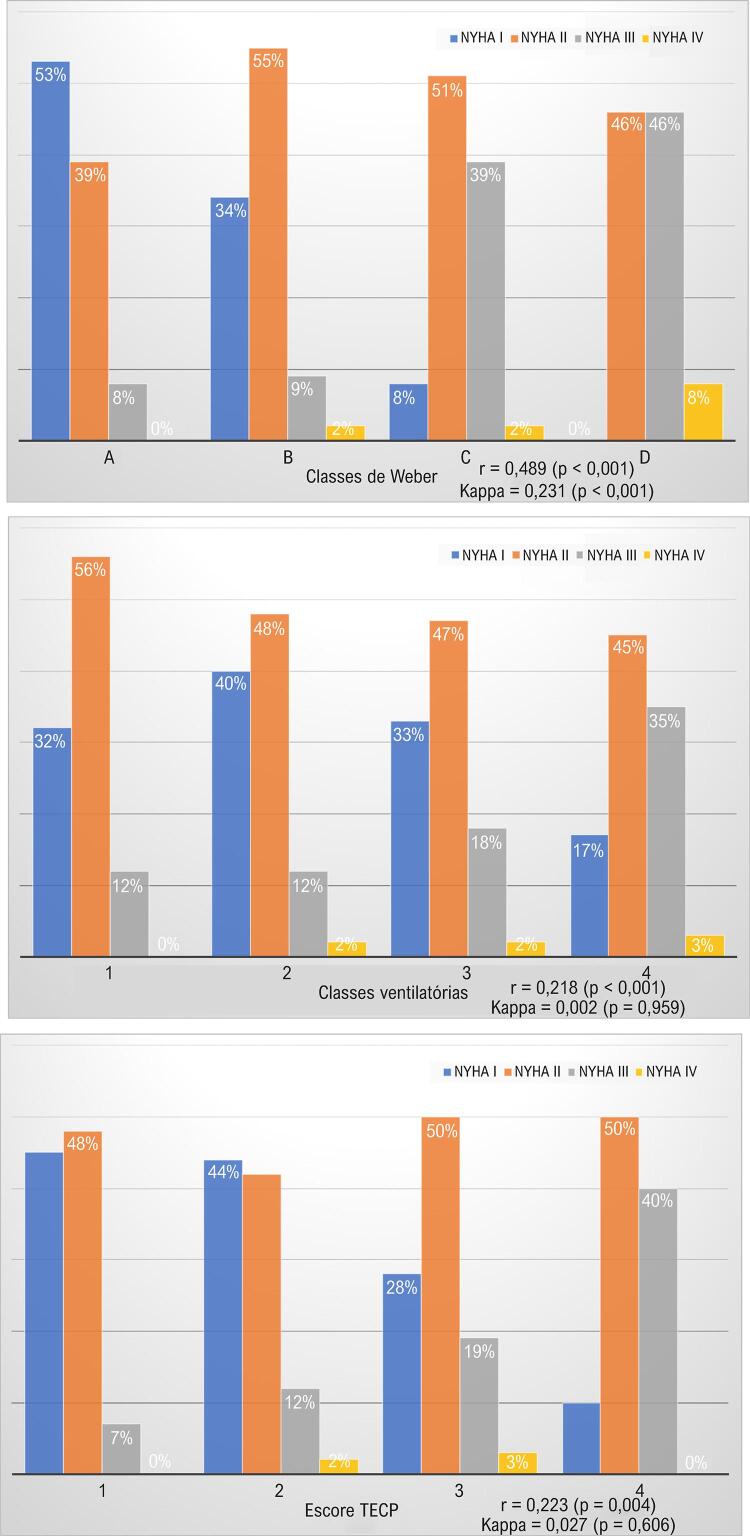
– Concordância, correlação e distribuição da classe da NYHA de acordo com as classes (A) de Weber, (B) ventilatórias (inclinação VE/VCO2) e (C) do escore TECP. TECP: teste de exercício cardiopulmonar; NYHA: New York Heart Association; r: coeficiente de correlação; VE/VCO2: v entilação minuto/produção de dióxido de carbono.

A Figura 1 mostra a distribuição das classes da NYHA de acordo com as classes de Weber ( Figura 1A ), ventilatórias ( Figura 1B ) e do escore TECP ( Figura 1C ). A correlação (r) entre as classes da NYHA e de Weber foi de 0,489 (p < 0,001), e a concordância foi de 0,231 (p < 0,001). A correlação (r) entre as classes da NYHA e ventilatórias foi de 0,218 (p < 0,001), e a concordância foi de 0,002 (p = 0,959). Por fim, a correlação (r) entre as classes da NYHA e do escore TECP foi de 0,223 (p = 0,004), e a concordância foi de 0,027 (p = 0,606).

## Discussão

Em pacientes com IC e FE reduzida que foram submetidos a TECP após indicação clínica, foi identificada apenas uma associação moderada entre as classes da NYHA e de Weber, com baixa concordância. No entanto, foram identificadas associação e concordância ainda mais baixas entre a classificação da NYHA e as classes ventilatórias e do escore TECP.

Todas essas classificações do estado funcional possuem um valor prognóstico validado para IC.^3 , 11^ Assim, o estado funcional é o melhor parâmetro para a predição de risco nesses pacientes.^3 , 11^ No entanto, conforme demonstrado, houve baixa concordância entre a classificação da NYHA e as três classificações baseadas no TECP (o qual é um teste clínico objetivo). Embora tenha sido encontrada uma correlação moderada entre as classes da NYHA e de Weber, parece razoável levantar a hipótese de que a subjetividade interfere na predição de risco para IC da classe da NYHA e apresenta um impacto subsequente nas decisões terapêuticas.

Uma recente revisão sistemática abordou a correlação entre a classificação da NYHA para IC e as medidas do VO_2peak_ (determinadas pelo TECP).^14^ Foi identificada uma grande heterogeneidade nas classes da NYHA entre os estudos analisados.^14^ Nossos achados corroboram os de Lim et al. e refletem uma análise de correlação adicional, visto que descrevemos a correlação entre a classificação subjetiva da NYHA e algumas classificações objetivas baseadas nos resultados do TECP através de escore validado ou classes ventilatórias. Por exemplo, os pacientes subjetivamente classificados na classe I da NYHA pelos seus médicos podem apresentar valores de inclinação VE/VCO_2_ classe ventilatória IV (pior prognóstico) ou se encontrar no pior quartil prognóstico do escore TECP ( Figura 1 ).^11 , 13^

A classificação da NYHA pode levar a diferentes interpretações do mesmo paciente por diferentes médicos,^3^ especialmente quando são relatados sintomas de classes intermediárias (II e III). Em uma publicação do nosso grupo, Ritt et al. demonstraram que os pacientes na classe B de Weber poderiam ser divididos em dois grupos prognósticos distintos quando o escore TECP era calculado.^15^ Os grupos foram, então, divididos em de maior risco e menor risco. No entanto, os pacientes nas classes intermediárias da NYHA geralmente são aqueles cujo estado funcional é de grande importância para a tomada de decisão. Essas decisões incluem o aumento ou a alteração de medicamentos, a provisão de indicações cirúrgicas ou a implantação de dispositivos (como terapia de ressincronização cardíaca ou dispositivo de assistência ventricular).^16^ Em tais grupos, a classificação da NYHA pode não ser sensível o suficiente para abordar características clínicas secundárias, mas importantes. Dessa forma, necessita-se urgentemente de uma classificação confiável, objetiva e de fácil reprodução. Os pacientes nas classes I ou II da NYHA podem ser reclassificados pelo TECP como de maior risco, e pacientes na classe III da NYHA podem ser reclassificados como de menor risco, principalmente aqueles que são candidatos para alterações de medicamentos e/ou dispositivos. O uso do TECP para esse objetivo é um tema para futuros estudos.

Nosso estudo apresenta algumas limitações, como a ausência de seguimento clínico da amostra de pacientes. Excluímos a anemia sintomática, visto que focamos em critérios diagnósticos clínicos, mas pode-se argumentar que a anemia assintomática também possa ter impacto na capacidade funcional. Além disso, não avaliamos a prevalência de depressão entre os pacientes, embora possa contribuir para a falta de esforço. Nossa amostra apresentou uma RER média de 1,04; pode-se argumentar que uma RER > 1,10 é o padrão para atingir a acidose, embora uma RER > 1,00 seja usada como um critério aceitável na IC.^17^ Embora isso possa afetar o VO_2peak_, não impacta a inclinação VE/VCO_2_, a OUES ou a HRR_1_. Novos estudos abordando uma população mais ampla e analisando desfechos clínicos são necessários para uma melhor compreensão do real valor prognóstico de cada classificação da IC (NYHA, inclinação VE/VCO_2_, classes de Weber e escore TECP). Focamos nas classes de Weber, nas classes da inclinação VE/VCO_2_ e no escore TECP porque todos esses parâmetros podem ser apresentados como classificações de escala de quatro níveis, como a NYHA; além disso, o VO_2peak_ e a inclinação VE/VCO_2_ são as variáveis mais estudas no TECP, e as outras variáveis do TECP estão inseridas no escore TECP. No entanto, estudos futuros com foco em variáveis específicas do TECP são de grande valor. É importante ressaltar que ainda resta determinar se, de fato, uma estratégia objetiva baseada no TECP é mais acurada do que as outras.

## Conclusão

Foi identificada uma associação moderada entre a classificação subjetiva da NYHA e as classes de Weber avaliadas objetivamente, embora a concordância tenha sido baixa. As classes ventilatórias avaliadas objetivamente e as classes do escore TECP apresentaram uma associação fraca e uma concordância baixa com a classificação da NYHA.
