# Supplementary material for: Baixa Concordância entre a Classificação da NYHA e as Variáveis do Teste de Exercício Cardiopulmonar em Pacientes com Insuficiência Cardíaca e Fração de Ejeção Reduzida
Source: Arq Bras Cardiol. 2022 Mar 21;118(6):1118–23. [Article in Portuguese] doi: 10.36660/abc.20210222 (PMC9345141; doi:10.36660/abc.20210222)
Supplement: Translation

# Translation: Low Concordance between NYHA Classification and Cardiopulmonary Exercise Test Variables in Patients with Heart Failure and Reduced Ejection Fraction

**Authors:** Luiz Eduardo Fonteles Ritt, Rebeca Sadigursky Ribeiro, Isabela Pilar Moraes Alves de Souza, João Victor Santos Pereira Ramos, Daniel Sadigursky Ribeiro, Gustavo Freitas Feitosa, Queila Borges de Oliveira, Ricardo Stein, Eduardo Sahade Darzé

**Affiliations:** 1 Brazil Instituto D’or de Pesquisa e Ensino (IDOR), Hospital Cárdio Pulmonar, Salvador, BA - Brazil; 2 Brazil Escola Bahiana de Medicina e Saúde Pública, Salvador, BA - Brazil; 3 Brazil Hospital de Clínicas de Porto Alegre, Universidade Federal do Rio Grande do Sul, Porto Alegre, RS - Brazil

**Keywords:** Heart Failure, Prognosis, Exercise Test

## Abstract

**Background:**

The New York Heart Association (NYHA) functional classification is the most commonly used classification system for heart failure (HF), whereas cardiopulmonary exercise testing (CPET) is the gold standard for functional status evaluation in HF.

**Objective:**

This study aimed to analyze correlation and concordance between NYHA classes and CPET variables.

**Methods:**

HF patients with clinical indication for CPET and ejection fraction (EF) < 50% were selected. Correlation (Spearman coefficient) and concordance (kappa) between NYHA classification and CPET-based classifications were analyzed. A p < 0.05 was accepted as significant.

**Results:**

In total, 244 patients were included. Mean age was 56 ± 14 years, and mean EF was 35.5% ± 10%. Distribution of patients according to NYHA classification was 31.2%% class I, 48.3% class II, 19.2% class III, and 1.3% class IV. Correlation (r) between NYHA and Weber classes was 0.489 (p < 0.001), and concordance was 0.231 (p < 0.001). Correlation (r) between NYHA and ventilatory classes (minute ventilation/carbon dioxide production [VE/VCO_2_] slope) was 0.218 (p < 0.001), and concordance was 0.002 (p = 0.959). Spearman correlation between NYHA and CPET score classes was 0.223 (p = 0.004), and kappa concordance was 0.027 (p = 0.606).

**Conclusion:**

There was a moderate association between NYHA and Weber classes, although concordance was low. Ventilatory (VE/VCO_2_slope) and CPET score classes had a weak association and a low concordance with NYHA classes.

## Introduction

Despite being a progressive disease, heart failure (HF) does not have a linear course. Hospitalizations due to HF decompensations are independent factors for prognosis. Risk prediction models and prognostic scores will determine the need to escalate specific therapeutic strategies, such as medication change, cardiac resynchronization therapy, implantable cardioverter-defibrillator, ventricular assist device, and cardiac transplantation.^1^

The New York Heart Association (NYHA) classification is a well-known, low-cost, simple functional stratification tool for HF with prognostic value.^2 , 3^ It divides patients into 4 different groups according to self-reported dyspnea severity and limitations to physical activities.^2 , 3^ However, the NYHA functional class depends on self-reported symptoms and, therefore, is influenced by the subjectivity of each patient.^4 , 5^

Conversely, functional status is assessed objectively by cardiopulmonary exercise testing (CPET), which is a prognostic tool considered to be the gold standard for HF assessment.^6 , 7^ Important guidelines define CPET as a class I recommendation for cardiac transplantation and a class IIa recommendation for exercise prescription in this context.^6 , 7^

Classically, CPET prognostic evaluation is based on peak oxygen uptake (VO_2peak_) measures.^8 , 9^ However, other variables such as minute ventilation/carbon dioxide production (VE/VCO_2_) slope, heart rate recovery in 1 minute (HRR_1_), oxygen uptake efficiency slope (OUES), end-tidal carbon dioxide partial pressure (PetCO_2_), and periodic ventilation have demonstrated an independent and incremental prognostic value to VO_2peak_in HF.^10^ Based on those variables, specific prognostic classifications have been validated, namely Weber classes (VO_2peak_), ventilatory classes (VE/VCO_2_slope), and CPET score (combining VO_2peak_, VE/VCO_2_slope, HRR_1_, OUES, and PetCO_2_).^11 - 13^

Even though the NYHA classification system is widely used, there are few studies correlating NYHA classes with HF prognosis or CPET variables.^14 , 15^ Recently, a systematic review compared NYHA classification and CPET variables, and the variable that was common to all analyzed studies was VO_2peak_, yet with much heterogeneity.^14^ This study aimed to evaluate correlation and concordance between NYHA classification for HF and CPET-based functional classifications, namely Weber classes, ventilatory classes, and CPET score.^11^

## Methods

This cross-sectional study consecutively recruited patients who underwent CPET for HF evaluation. Inclusion criteria were the following: 1) age ≥ 18 years; 2) confirmed HF diagnosis with ejection fraction (EF) < 50%; and 3) clinical indication for CPET between 2009 and 2019. Exclusion criteria were moderate-to-severe chronic obstructive pulmonary disease, pulmonary hypertension, and/or fibrosis or symptomatic anemia.

CPET variables and demographic data were collected together with clinical information and relevant complementary tests (12-lead resting electrocardiogram and Doppler echocardiogram from the past 3 months). CPET was symptom-limited and was performed at maximal effort with a ramp protocol in a treadmill (Micromed Centurion 300, São Paulo, Brazil) using a Cortex 3b breath-by-breath analyzer (Cortex Inc., Leipzig, Germany). Two-point gas calibration was done before the tests. All techniques followed current guidelines, and a nationally certified physician was responsible for each test.^10^

All CPET tests were conducted by the same physician, a cardiologist who specializes in CPET. Before CPET, the same physician in charge of the test determined each patient’s NYHA class according to self-reported limitation to physical activity: (I) no limitation to physical activity; (II) slight limitation to physical activity; (III) marked limitation to physical activity; or (IV) unable to perform any physical activity without discomfort.^16^ Then, based on CPET variables, patients were classified into Weber classes, ventilatory classes, and CPET score classes according to their CPET results.^11^

Weber classification categorizes patients according to their VO_2peak_as follows: (A) VO_2_> 20 mL.kg^-1^.min^-1^; (B) VO_2_16-20 mL.kg^-1^.min^-1^; (C) VO_2_10-15 mL.kg^-1^.min^-1^; or (D) VO_2_< 10 mL.kg^-1^.min^-1^.^12^ Ventilatory classes use VE/VCO_2_slope: (I) VE/VCO_2_≤ 29.9; (II) VE/VCO_2_30-35.9; (III) VE/VCO_2_36-44.9; or (IV) VE/VCO_2_≥ 45.^13^ CPET score was calculated for each patient based on the summation of abnormal responses as follows: VE/VCO_2_≥ 34 (7 points); HRR_1_≤ 6 bpm (5 points); OUES ≤ 1.4 (3 points); PetCO_2_< 33 mm Hg (3 points); and VO_2peak_≤ 14 mL.kg^-1^.min^-1^(2 points).^11 , 15^ The score is then divided into quartiles: (I) 0-5; (II) 6-10; (III) 10-15; and (IV) > 15.^11^

### Statistical analysis

SPSS version 17.0 (SPSS Inc., Chicago, IL, USA) was used for all statistical analyses. Continuous variables were reported as mean and standard deviation for parametric distribution or as median and interquartile range for nonparametric distribution. Kolmogorov-Smirnov normality test and histogram analysis were used for determination of distribution. Categorical variables were reported as absolute numbers and proportions. Correlation between variables was assessed using Spearman ( *s* ) or Pearson ( *p* ) correlation coefficient, and concordance was assessed using kappa (k) coefficient. For all analyses, a p < 0.05 was accepted as statistically significant.

An institutional research ethics committee approved the study protocol. Also, the study respects all national and international regulations for human research.

## Results

Patients’ characteristics are described in Table 1 . The sample included 244 patients, mainly men (77.9%), and mean age was 56 ± 14 years. Ischemia was the most frequent etiology (44.4%). Mean EF was 35.5% ± 10%. Patients were on optimized medical therapy as follows: 86.4% angiotensin-converting enzyme inhibitors or angiotensin II receptor blockers, 91.4% beta-blockers, 57.0% aldosterone antagonists, and 53.5% diuretics. Mean VO_2peak_was 19.2 ± 6.7 mL.kg^-1^.min^-1^, whereas mean VE/VCO_2_slope was 39 ± 10. Mean respiratory exchange ratio (RER) was 1.041 ± 0.12 (25% had a RER > 1.10). All tests were interrupted by the effort criteria, and none was interrupted prematurely or due to hemodynamic, arrhythmic, or ischemic criteria. Patients were distributed according to NYHA classification as follows: 31.3% class I, 48.3% class II, 19.2% class III, and 1.3% class IV ( Table 2 ).

**Table 1:** – General patient demographic, clinical, and cardiopulmonary exercise test characteristics (n = 244)

Variables	
**Age** (mean ± SD)	56±14 years
**Gender**	
Male, n (%)	190 (77.9)
**Etiology**	
Ischemic, n (%)	107 (44.4)
Idiopathic, n (%)	56 (23.2)
Viral, n (%)	30 (12.4)
Chagasic, n (%)	18 (7.5)
Other, n (%)	30 (12.5)
**Comorbidities**	
Hypertension, n (%)	70 (34.7)
Diabetes mellitus, n (%)	43 (21.2)
Coronary artery disease, n (%)	94 (46.3)
Smoking, n (%)	4 (2.0)
**Medications used**	
ACEI or ARB, n (%)	209 (86.4)
Beta-blocker, n (%)	222 (91.4)
MCRA, n (%)	138 (57.0)
Diuretics, n (%)	129 (53.5)
**Implantable devices**	
Pacemaker, n (%)	17 (7.0)
CRT and/or ICD, n (%)	28 (11.5)
**VO**_**2peak**_ **(mL.kg** ^**-1**^ **.min** ^**-1**^ **),** mean ± SD	19.2±6.7
**Percent of predicted VO**_**2peak**_ **(%),** mean ± SD	63±20
**EF (%),** mean ± SD	35.5±10
**RER,** mean ± SD	1.041±0.12
**VE/VCO**_**2**_ **slope,** mean ± SD	39.0±10.8
**PetCO**_**2**_ **(mm Hg),** mean ± SD	29.8±4.66
**HRR**_**1**_ , median (IQR)	18.0 (15)
**SBP at rest,** median (IQR)	120 (10)
**HR at rest,** median (IQR)	74 (22)

*ACEI: angiotensin-converting enzyme inhibitor; ARB: angiotensin II receptor blocker; MCRA: mineralocorticoid-receptor antagonists; CRT: cardiac resynchronization therapy; ICD: implantable cardioverter-defibrillator; VO_2peak_: peak oxygen uptake; SD: standard deviation; EF: ejection fraction; RER: respiratory exchange ratio; VE/VCO_2_: minute ventilation/carbon dioxide production; PetCO_2_: end-tidal carbon dioxide partial pressure; HRR_1_: heart rate recovery in 1 minute; SBP: systolic blood pressure; HR: heart rate; NYHA: New York Heart Association; CPET: cardiopulmonary exercise test; IQR; interquartile range*

**Table 2:** – Sample distribution according to subjective and objective classifications, n (%)

	I	II	III	IV
NYHA class	75 (31.2)	116 (48.3)	46 (19.2)	3 (1.3)
VE/VCO_2_slope	42 (17.2)	70 (28.7)	74 (30.3)	58 (23.8)
CPET score	57 (34.7)	61 (37.2)	36 (22.0)	10 (6.1)
	A	B	C	D
Weber class	95 (39)	55 (22.5)	81 (33.2)	13 (5.3)

*NYHA: New York Heart Association; VE/VCO_2_: minute ventilation/carbon dioxide production; CPET: cardiopulmonary exercise test.*

Figure 1 shows NYHA class distribution according to Weber classes ( Figure 1A ), ventilatory classes (Figure 1B), and CPET score classes (Figure 1C). Correlation (r) between NYHA and Weber classes was 0.489 (p < 0.001), and concordance was 0.231 (p < 0.001). Correlation (r) between NYHA and ventilatory classes was 0.218 (p < 0.001), and concordance was 0.002 (p = 0.959). Finally, correlation (r) between NYHA and CPET score classes was 0.223 (p = 0.004), and concordance was 0.027 (p = 0.606).

**Figure 1:** – NYHA class distribution, correlation, and concordance according to (A) Weber classes, (B) ventilatory classes (VE/VCO2 slope), and (C) CPET score classes. NYHA: New York Heart Association; VE/VCO2: minute ventilation/carbon dioxide production; CPET: cardiopulmonary exercise test; r: correlation coefficient.

## Discussion

In patients with HF with reduced ejection fraction who underwent CPET after clinical indication, we found only a moderate association between NYHA and Weber classes, with a low concordance. However, there was an even lower association or concordance rate between NYHA classification and ventilatory or CPET score classes.

All those functional status classifications have their prognostic value validated for HF.^3 , 11^ Thus, functional status is the best parameter for risk prediction in those patients.^3 , 11^ However, as we showed, there was a low concordance between NYHA classification and the 3 classifications based on CPET (which is an objective clinical test). Even though we found a moderate correlation between NYHA and Weber classes, it seems reasonable to hypothesize that subjectivity interferes in NYHA classification risk prediction for HF and has a subsequent impact on therapeutic decisions.

A recent systematic review addressed the correlation between NYHA classification for HF and VO_2peak_measures (determined by CPET).^14^ It found a great heterogeneity in NYHA classes among the included studies.^14^ Our findings support those of Lim et al. and reflect a further correlation analysis, as we described the correlation between the subjective NYHA classification and some objective classifications that are based on CPET results, either through a validated score or through ventilatory classes. For example, patients subjectively considered to be in NYHA class I by their attending physicians may have ventilatory class IV VE/VCO_2_slope values (poorest prognosis) or be in the poorest prognostic quartile of the CPET score ( Figure 1 ).^11 , 13^

NYHA classification may lead to different interpretations of the same patient from different attending physicians,^3^ especially when symptoms from intermediate classes (II and III) are reported. In a publication from our group, Ritt et al. demonstrated that patients in Weber class B could be divided into two different prognostic groups when the CPET score was calculated.^15^ The groups were then divided into one of higher risk and another of lower risk. However, patients in intermediate NYHA classes are generally those whose functional status is of great importance for decision-making. These decisions include increasing or changing medications, providing surgical indications, or implanting devices (such as cardiac resynchronization therapy or ventricular assist device).^16^ In such groups, NYHA classification may not be sensitive enough to address minor but important clinical features. Therefore, an objective, easily reproducible, reliable classification is urgently needed. In patients with NYHA class I or II, CPET may reclassify them to higher risk, and patients with NYHA class III may be reclassified to lower risk, especially those who are candidates to medication changes and/or devices. The use of CPET for this purpose is a matter for future studies.

Our study has some limitations, such as lack of clinical follow-up of our patient sample. We excluded symptomatic anemia, as we focused on clinical diagnostic criteria, but one may argue that asymptomatic anemia may also impact functional capacity. Also, the prevalence of depression was not assessed in our patients, although it may contribute to the lack of effort. Our sample had a mean RER of 1.04; one may argue that a RER > 1.10 is the pattern for achieving acidosis, although in HF some use RER > 1.00 as an acceptable criterion.^17^ Although this may impact VO_2peak_, it does not impact VE/VCO_2_slope, OUES, or HRR_1_. New studies addressing a wider population and analyzing clinical outcomes are necessary to a better understanding of the actual prognostic value of each HF classification (NYHA, VE/VCO_2_slope, Weber classes, and CPET score). We focused on Weber classes, VE/VCO_2_slope classes, and CPET score because all these parameters may be presented as 4-level scale classifications as NYHA; also, VO_2peak_and VE/VCO_2_slope are the most studied variables in CPET, and other variables from CPET are inserted in the CPET score. However, future studies focusing on specific CPET variables are valuable. Importantly, it remains to be determined whether there is, in fact, an objective CPET-based strategy that is more accurate than the others.

## Conclusion

There was a moderate association between the subjective NYHA classification and the objectively measured Weber classes, although concordance was low. The objectively measured ventilatory classes and CPET score classes had a weak association and a low concordance with the NYHA classification.
